# Using Over-the-Counter Retail Medication Sales to Detect and Track Influenza-Like Illnesses Including Novel Diseases: Surveillance Modeling Study

**DOI:** 10.2196/91163

**Published:** 2026-10-21

**Authors:** Ye Ye, Jessi Espino, John M Aronis, Harry Hochheiser, Marian G Michaels, Gregory F Cooper

**Affiliations:** 1 Department of Biomedical Informatics, University of Pittsburgh, 3420 Forbes Avenue, 8th Floor Murdoch Building, Pittsburgh, PA, 15213, United States, 1 4129252746; 2 UPMC Children's Hospital of Pittsburgh, Pittsburgh, PA, United States

**Keywords:** public health surveillance, over-the-counter medication sales, emergency department visits, influenza-like illness, novel diseases, disease outbreak detection, anomaly detection, probabilistic modeling, expectation-maximization algorithm

## Abstract

**Background:**

Traditional disease surveillance systems often rely on clinical or laboratory-confirmed data, which may delay detection of emerging outbreaks. The National Retail Data Monitor (NRDM), established in 2002, collects anonymized over-the-counter (OTC) health product sales data from more than 28,000 retail outlets across the United States and provides a complementary data source for syndromic surveillance.

**Objective:**

This study evaluated the use of NRDM data for detecting and tracking influenza-like illness (ILI), including purchasing patterns suggestive of novel disease outbreaks.

**Methods:**

NRDM data from Allegheny County, Pennsylvania, collected between 2016 and 2021, were analyzed using a probabilistic modeling framework to estimate daily ILI activity from OTC purchasing patterns. An expectation-maximization algorithm was applied to data from the training period (June 2016-May 2019) to estimate conditional probabilities of OTC product purchases under ILI and non-ILI conditions. These estimates were then applied to daily sales data from the test period (June 2019-December 2021) to infer daily ILI counts. The framework was further extended into a monitoring system, termed NRDM Tracker, which uses daily log-likelihoods and false discovery rate–adjusted *P* values to detect statistically significant anomalies in purchasing behavior.

**Results:**

NRDM-derived ILI estimates were moderately correlated with emergency department (ED)–based ILI counts (*r*=0.66). Compared with other OTC product categories (eg, thermometers and antifever medications), cough and cold medications showed the strongest association with ILI-related purchasing behavior and higher estimated purchase probability under ILI conditions (0.792) than under non-ILI conditions (0.695). During the early phase of the COVID-19 pandemic in March 2020 in Allegheny County, when COVID-19 testing capacity was limited and public awareness and health-seeking behaviors were rapidly evolving, NRDM-based ILI signals increased before corresponding rises in confirmed ED-based COVID-19 counts. Moreover, NRDM Tracker identified significant anomalies in OTC purchasing behavior during major outbreak periods, including a pronounced decline in log-likelihood in March 2020 corresponding to the onset of the COVID-19 pandemic. Multiple product categories exhibited statistically significant deviations in sales during these periods.

**Conclusions:**

OTC product sales data, analyzed using probabilistic modeling and anomaly detection methods, can provide timely insights into population-level health activity and may help identify anomalies associated with emerging disease outbreaks. NRDM-based surveillance systems may complement traditional clinical surveillance and strengthen local and national biosurveillance capabilities.

## Introduction

Detection of disease outbreaks is critical to public health surveillance, particularly in the early identification of emerging outbreaks. Traditional surveillance approaches, such as sentinel systems operated by the Centers for Disease Control and Prevention (CDC), rely on confirmed case reports from selected health care systems. While these methods provide accurate clinical insights, they can suffer from reporting delays, limiting their effectiveness for timely response.

To support timely detection, public health surveillance has evolved to incorporate diverse data streams, including electronic health records (EHRs) [1-4], search engine queries [5,6], and social media [7,8], and participatory surveillance systems [9-14] in which individuals directly report symptoms through digital platforms. EHR-based surveillance enables real-time insights from structured health care data but depends on integration with clinical systems. Search engine–based surveillance leverages aggregated query trends, for example, spikes in Google searches for influenza symptoms, to estimate disease activity in near real time. Social media–based surveillance mines user-generated content, such as symptom-related posts, to detect unusual patterns in health behavior. In contrast, participatory surveillance relies on structured, self-reported data rather than passively collected online content. Despite their promise, these approaches—particularly those based on passively collected online data—can be sensitive to shifts in platform algorithms and user behavior, and their spatial resolution may be limited for local outbreak detection, which may reduce their reliability for long-term surveillance.

An additional, underused approach involves analyzing over-the-counter (OTC) product sales, such as thermometer sales, which can reflect self-treatment behaviors [15]. Since these purchases often precede formal medical care, anomalies in OTC sales can signal emerging population-level health events. To support early outbreak detection nationwide, the National Retail Data Monitor (NRDM) [16] was launched in December 2002 as a novel public health surveillance system. Operated by the University of Pittsburgh, the NRDM has collected anonymous sales data on selected OTC health care products, such as antipyretics (fever-reducing medications), cough and cold medications, and thermometers from more than 28,000 participating retail pharmacies, grocery stores, and mass merchandise outlets across the United States. The NRDM was developed to enhance public health capacity for syndromic surveillance, enabling rapid detection of unusual health events through deviations in consumer purchasing behavior. More than 800 public health officials in 49 states, the District of Columbia, and US territories have had access to NRDM through secure online portals [17], supporting them to track anomalies in consumer health behavior in near real time.

Since the launch of the NRDM, studies have been conducted to enhance its functionality from multiple perspectives. Methodological advances include the development of fast spatial and space-time scan algorithms and the integration of user feedback mechanisms [18]. Additionally, application-oriented studies have examined the correlation between OTC sales and health care use, including influenza-like illness (ILI) at urgent care centers [19]. Numerous studies have validated the value of NRDM in complementing traditional surveillance systems for investigations of gastrointestinal illness outbreaks—including those in the San Francisco Bay Area [20], and in New York City in the context of a massive power outage in August 2003 [21]. NRDM data have also been used to help detect spikes in unintentional poisonings [22], support syndromic surveillance during the San Diego wildfires in 2003 [23], and as a component of New York City’s syndromic surveillance systems [24].

We previously developed ILI Tracker [25], a Bayesian surveillance method that uses emergency department (ED) data to estimate the daily frequency of multiple ILIs, including potential novel diseases. As an illustrative example, retrospective analysis indicated that applying ILI Tracker to 2014 data would have enabled early detection of an enterovirus D68 outbreak in Allegheny County [25].

Building on this foundation, we sought to determine whether prediagnostic, population-level signals—specifically, OTC product purchases—could support outbreak detection. To this end, we developed and evaluated a similar probabilistic modeling system that uses NRDM data to detect outbreaks of known ILI diseases. The system also seeks to identify unusual purchasing patterns that may indicate the onset of novel diseases by detecting statistically significant deviations in OTC product purchases relative to expected purchases for known disease models. We call this system NRDM Tracker. We evaluated the NRDM Tracker system on five years of real OTC data in Allegheny County.

The objectives of this study were to (1) evaluate whether OTC purchasing patterns can be used to estimate population-level ILI activity, (2) assess the agreement between NRDM-derived ILI estimates and ED-based ILI surveillance measures, and (3) determine whether anomaly detection methods applied to OTC sales data can identify unusual purchasing patterns associated with emerging disease outbreaks. We hypothesized that OTC purchasing patterns contain sufficient information to estimate population-level ILI activity and that model-derived ILI estimates based on OTC sales data would track temporal changes in population-level ILI activity well. We further hypothesized that OTC sales data would be correlated with ED-based ILI activity and that significant deviations from expected purchasing patterns could provide early signals of novel disease outbreaks.

## Methods

### Research Data

We retrieved OTC product sales for Allegheny County, Pennsylvania (270 stores) from the NRDM database, covering the period from June 1, 2016, to December 30, 2021. NRDM product codes were aggregated into 7 analytically defined OTC categories (Table 1). The full time series was partitioned into a training interval (June 1, 2016-May 31, 2019) and a held-out test interval (June 1, 2019-December 30, 2021). The training interval was used exclusively for model parameter estimation (refer to the *Model Development* section), whereas the test interval was reserved for out-of-sample model evaluation and prospective abnormal event detection (refer to the subsequent sections under *Methods*).

**Table 1.** Seven over-the-counter (OTC) product categories derived from National Retail Data Monitor (NRDM) sales data collected from 270 retail stores in Allegheny County, Pennsylvania, United States (June 2016–December 2021).

Category	Description
Cough or cold	All cough and cold related medications, including pediatric and adult formulations of liquids and tablets, bronchial remedies, chest rubs, nasal sprays, and throat lozenges.
Antifever adult	All products intended for adults that are taken orally for the relief of fever
Antifever pediatric	All products intended for children that are taken orally for the relief of fever
Baby or child electrolyte	Solutions of primarily electrolytes and glucose used for fluid replacement in children with dehydration; includes products in liquid and freezer pop form
Thermometer	All thermometers, designed for both pediatric and adult use, including electronic and “ear” (also known as tympanic membrane) thermometers used for health reasons; also includes nonintrusive fever detectors and replacement sheaths for electronic thermometers
Antidiarrheal	Products taken for the relief of diarrhea; all sizes and forms (tablets, liquids, etc); includes bismuth (Pepto Bismol and like products), attapulgite, subsalicylate, and loperamide hydrochloride products
Hydrocortisone product	Anti-inflammatory topical corticosteroids used to relieve itching rashes

To support the method that we used for estimating parameters in the NRDM Tracker model, we derived the daily incidence rate of ILI in the population, which serves as the prior probabilities in the model. To do so, we obtained daily counts of ED encounters associated with ILI-related viruses in the region, including adenovirus, rhinovirus or enterovirus, influenza, human metapneumovirus (HMPV), parainfluenza (PIV), and respiratory syncytial virus (RSV). For each day, we calculated the proportion of encounters related to ILI among all ED encounters. ILI counts were identified based on either positive laboratory test results or relevant *ICD* (*International Classification of Diseases*) diagnosis codes (Multimedia Appendix 1). The participating EDs included (1) University of Pittsburgh Medical Center (UPMC) Presbyterian University Hospital, (2) UPMC Children’s Hospital of Pittsburgh, (3) UPMC Shadyside, (4) McKeesport Hospital, and (5) UPMC Mercy Hospital. These 5 sites collectively represent a significant fraction (about 60%) of ED visits in Allegheny County and 30%‐40% of western Pennsylvania. The research data were sourced from the University of Pittsburgh Neptune Research Data Warehouse [26].

The NRDM and ED datasets share strong geographic and population overlap, as both focus on Allegheny County, Pennsylvania. We retrieved OTC sales data from 270 retail stores across the county, while the 5 EDs used for estimating prior disease probabilities also serve this region. The coverage of the NRDM for the Pittsburgh metropolitan statistical area, including Allegheny County, is 73% of all retail stores in the area. Although the datasets are not individually linked, their shared regional focus supports the use of ED-based ILI daily incidence estimates in helping model NRDM purchasing patterns.

### Model Development

Since many OTC products are used to relieve symptoms rather than treat specific diseases, we grouped conditions into two broad categories: (1) ILI diseases and (2) all other diseases, which we label as OTHER. The main abbreviations we used to describe our methods are summarized in Table 2.

**Table 2.** Mathematical notation used in the NRDM^a^ tracker probabilistic surveillance model for estimating influenza-like illness activity from OTC^b^ product sales.

Symbols	Descriptions
D	All OTC sales data during the surveillance time period
D(p)	A single OTC product bought by a customer with purchase *p*; if a customer buys multiple products in one transaction, each product is treated as a separate purchase. D(p)∈{cough or cold,antifever for adult,antifever for pediatric,baby or child electrolytes,thermometers,antidiarrheal,hydrocortisones}
$pur_{d}$	Total number of OTC purchases on day *d*
$P_{d}\left(D\right)$	Probability of the observed data on day *d*
$P_{d}\left(D\left(p\right)\right)$	Probability of an individual purchase p on day d
$dx_{i}$	Disease group *i*; *dx*_1_ = influenza-like illness (ILI); *dx*_2_ = diseases other than ILI (OTHER)
$P_{d}\left(D\left(p\right),dx_{i}\right)$	The joint probability that a customer with a disease in group $dx_{i}$ makes purchase p on day d
$P_{d}\left(D\left(p\right)|dx_{i}\right)$	The conditional probability that a customer makes purchase p on day d, given they have a disease in group $dx_{i}$
$P_{d}\left(dx_{i}\right)$	Prior probability that an OTC-purchasing customer has a disease in group *i* on day *d*
$P_{d}\left(dx_{i}|D\left(p\right)\right)$	The posterior probability that a customer has a disease in group $dx_{i}$ given that they make purchase p on day d.

a NRDM: National Retail Data Monitor.

b OTC: over-the-counter.

As described further in this study, we applied the expectation-maximization (EM) algorithm [27] (refer to Algorithms 1 and 2 in Multimedia Appendix 2) to estimate the conditional probabilities $P_{d}\left(D\left(p\right)|dx_{i}\right)$, where $P_{d}\left(D\left(p\right)=product_{j}|dx_{i}=ILI\right)$ represents the probability a customer purchases product *j* on day *d* due to ILI either in that customer or in someone they are making the purchase for (eg, a family member). $P_{d}\left(D\left(p\right)=product_{j}|dx_{i}=OTHER\right)$ denotes the corresponding probability for patients with conditions other than ILI.

In applying EM to estimate the conditional probabilities above, we made the following assumptions:

1. Time invariance: The conditional probabilities are constant across days, that is, $P_{d}\left(product_{j}|dx_{i}\right)$ is the same for all *d*. This assumes that given a disease-group category (eg, ILI), the likelihood of purchasing a specific product remains stable over time. It is justified by the expectation that symptom-driven consumer behavior remains stable, especially when product availability and public health messaging are consistent. This assumption simplifies modeling by attributing temporal variation to disease-category daily incidence rather than to changes in individual purchasing behavior.
2. Product focusing: We only monitored the purchase of the product categories presented in Table 1.

The EM algorithm iteratively refines probability estimates through two main steps: the expectation step (E-step) and the maximization step (M-step).

In the E-step, the algorithm computes the posterior probabilities $P_{d}\left(ILI|product_{j}\right)$ and $P_{d}\left(OTHER|product_{j}\right)$ for each product *j* on day *d*, using the current estimates of $P\left(product_{j}|ILI\right)$ and $P\left(product_{j}|OTHER\right)$, along with the prior probabilities of ILI and OTHER on day *d* estimated from ED data (refer to details in the *Research Data* section).

In the M-step, the algorithm updates the conditional probabilities, $P\left(product_{j}|ILI\right)$ and $P\left(product_{j}|OTHER\right)$ by maximizing the expected likelihood. This is achieved by leveraging the weighted counts of product *j* purchases on day *d*, denoted as, $N_{d}^{j}$, using the posterior probabilities obtained from the E-step.

The E-step and M-step are repeated iteratively until convergence is achieved—that is, until the maximum change in estimates of the 2 conditional probabilities falls below a predefined threshold, *tol*.

We used ED data in the Allegheny County region to estimate the priorprobabilities, $P_{d}\left(dx_{i}\right),$ used by the EM algorithm. To smooth the ED time series, we applied a centered 7-day moving average to the daily encounter counts. At the beginning and end of the series, where fewer neighboring values were available, a smaller window size (eg, 4 or 5 days) was used to compute the average. This adaptive window ensures that smoothing can still be applied at the edges without discarding data. Based on the resulting smoothed counts, we calculated daily prior probabilities for ILI and OTHER that were used by the EM algorithm.

### Estimates of Daily Disease Counts Using NRDM Data

After estimating the conditional probabilities $P\left(product_{j}|dx_{i}\right)$, we derive the expected number of customers with disease group $dx_{i}$ on day *d*, denoted $E_{d}\left({dx}_{i}\right)$, using equation 1. Specifically, $E_{d}\left({dx}_{i}\right)$ is calculated as the sum of the posterior probabilities $P_{d}\left(dx_{i}|D\left(p\right)\right)$ across all purchases on day *d*. For each individual purchase *p*, the posterior probability is computed via Bayes' rule (equation 2), where the numerator is the joint probability, calculated as the product of the conditional probability of the purchase given the disease (as estimated via the EM algorithm) and the prior probability of the disease. The denominator normalizes this quantity by summing over the 2 disease group categories that we model.

In contrast to the EM training phase, where disease priors were learned from ED data, we fixed the priors at $P_{d}\left(ILI\right)=0.05$ and $P_{d}\left(OTHER\right)=0.95$ for each day during the calculation with equation 3 to focus the contribution of NRDM data in estimating daily disease patterns. These values are within the bounds of the probabilities for ILI and OTHER estimated from ED data during the training phase, during which the daily ILI daily incidence ranged from 0 to 0.06 and the daily OTHER daily incidence ranged from 0.94 to 1 (refer to the *Descriptive Statistics* section; Figure 1).

**Figure 1.**
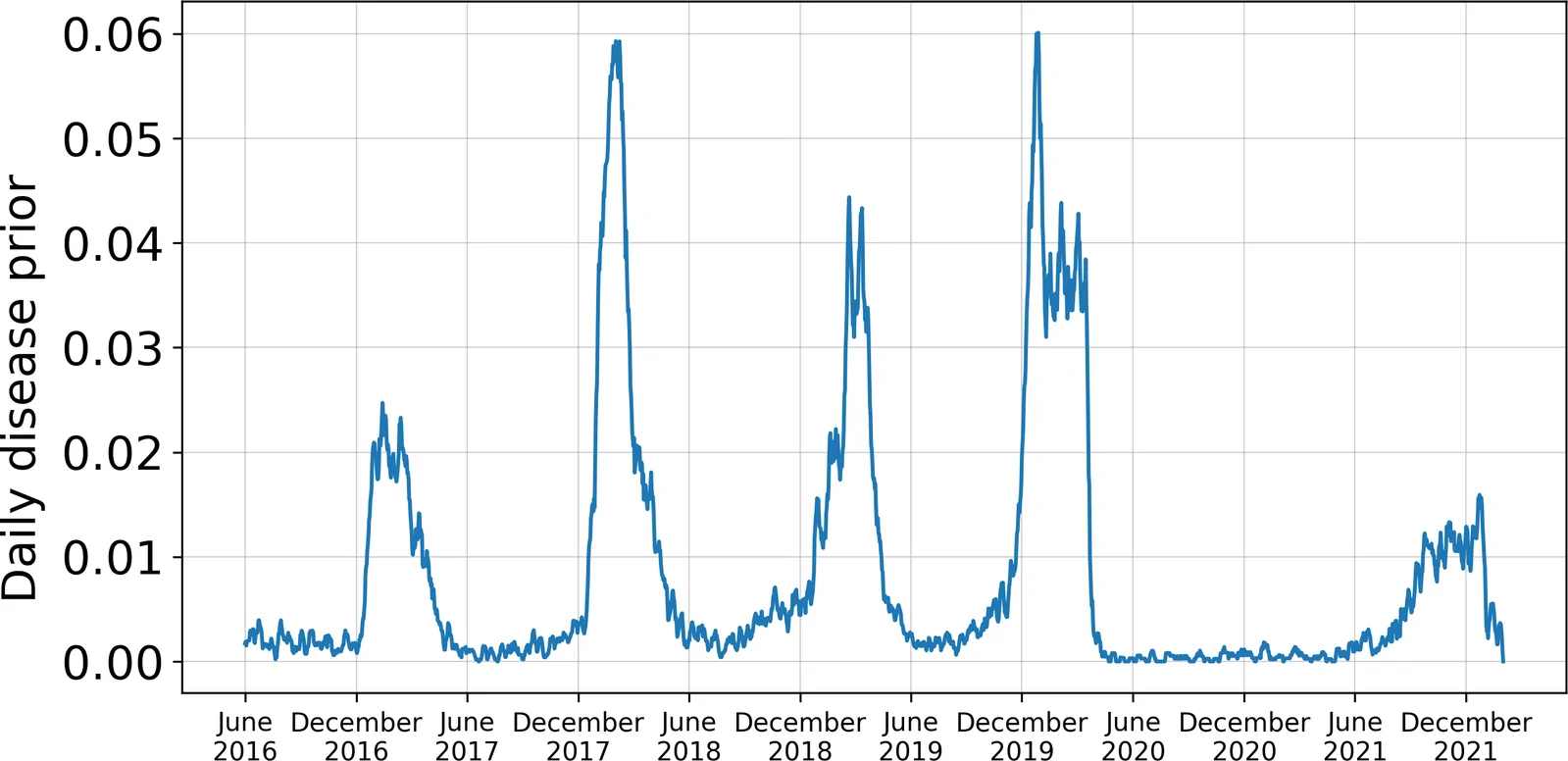
Estimated daily influenza-like illness (ILI) incidence rates derived from emergency department encounters at 5 hospitals in Allegheny County, Pennsylvania, United States, from June 2016 to December 2021 and used as disease priors in the NRDM Tracker model.


(1) $$E_{d}\left(dx_{i}\right)=\sum_{p=1}^{pur_{d}}P_{d}\left(dx_{i}|D\left(p\right)\right)$$

(2) $$P_{d}\left(dx_{i}|D(p)\right)=\frac{P_{d}\left(D(p)|dx_{i}\right)\times P_{d}\left(dx_{i}\right)}{\sum_{k=1}^{2}P_{d}\left(D(p)|dx_{k}\right)\times P_{d}\left(dx_{k}\right)}$$

### Derivation of the Likelihood of Daily Data

For each day *d*, we used a probabilistic metric, $P_{d}\left(D\right)$, as an indicator of the presence of unusual purchases on that day. This metric represents the probability of the observed data on day *d* (ie, the number and types of OTC purchases on that day). We refer to this probability as the *daily likelihood*. Using equation 3, we estimate this quantity by multiplying the probabilities of all individual purchase events $P_{d}\left(D\left(p\right)\right),$ assuming that these $pur_{d}$ purchase events are independent. A purchase behavior typically reflects a customer’s (or their household member’s) illness. However, the specific underlying disease is not recorded in the NRDM database. Therefore, for each purchase behavior, we sum the joint probabilities, $P_{d}\left(D\left(p\right),dx_{i}\right)$, across the 2 disease groups that we modeled.


(3) $$\begin{array}{rl} P_{d}(D) & =\prod_{p=1}^{pur_{d}}P_{d}\left(D(p)\right)\\ =\prod_{p=1}^{pur_{d}}\sum_{i=1}^{2}P_{d}\left(D(p),dx_{i}\right)\\ =\prod_{p=1}^{pur_{d}}\sum_{i=1}^{2}P_{d}\left(D(p)|dx_{i}\right)P_{d}\left(dx_{i}\right)\\ =\prod_{p=1}^{pur_{d}}\left[P_{d}\left(D(p)|ILI\right)P_{d}\left(ILI\right)+P_{d}\left(D(p)|OTHER\right)P_{d}\left(OTHER\right)\right]. \end{array}$$

Each joint probability is computed as the product of the prior probability of disease ${dx}_{i}$ on day *d*, denoted as $P_{d}\left({dx}_{i}\right)$, and the conditional probability $P_{d}\left(D\left(p\right)|dx_{i}\right)$, which is the probability of observing the purchase behavior given the presence of disease group ${dx}_{i}$.

To estimate these conditional probabilities in the model training period, we applied the EM algorithm, as discussed in the *Model Development* section. As mentioned, when training the NRDM Tracker model, we used the daily incidence of diseases in EDs in the same region on day *d* to estimate the prior probability, $P_{d}\left(dx_{i}\right)$. When applying the model to monitor for disease outbreaks, we used a uniform prior for $P_{d}\left(dx_{i}\right)$, $P_{d}\left(ILI\right)=0.05$ , $P_{d}\left(OTHER\right)=0.95$, which does not require having access to ED data.

### Derivation of a Normalized Likelihood of Daily Data

We also derive the following normalized likelihood (**Equation 4**), which is the geometric mean of the daily likelihood across all purchases on day *d*:


(4) $$G_{d}\left(D\right)=\sqrt[{pur_{d}}]{P_{d}\left(D\right)},$$

where $pur_{d}$ is the total number of the products purchased in the 7 OTC categories on day *d*. $G_{d}\left(D\right)$ reflects the (geometric) average likelihood of individual product purchases. Changes in the unnormalized likelihood, $P_{d}\left(D\right),$ may be due to changes in the relative distribution of the products being sold, the number of products being sold, or both. In contrast, changes in $G_{d}\left(D\right)$ are only due to changes in the relative distribution of the products being sold.

### Detection and Characterization of a Putative Outbreak of a Novel Disease

This section describes the methods that NRDM Tracker applies to monitor a test set of NRDM data for disease outbreaks. It uses the log of the likelihood given by equation 3 to look for low values that may indicate an outbreak. When values are low, it can analyze the data at the product level to look for patterns that may indicate which product categories are being influenced by a putative outbreak; such a characterization may suggest the type of outbreak that is occurring. In the remainder of this section, we discuss how we derive *P* values at the product level.

For each product *j*, we obtain the daily sales count $n_{d}^{j}$ for day *d* and computed a right-sided *P* value based on the empirical distribution of daily sales during a control period from day *d*-38 to day *d*-8, assuming a normal distribution. Approximately 69% (4326/6284) of Shapiro-Wilk normality tests [28] passed the normality check, indicating that most control period count distributions were consistent with a normal distribution.

Since a *P* value is calculated for each day, over many monitored days, we applied the Benjamini-Hochberg procedure [29] to control the false discovery rate (FDR) at a significance level of 0.05. Specifically, we used the multiple tests function from the Python statsmodels.stats.multitest module (statsmodels 0.15.0, 2024) with the method=‘fdr_bh’ option to correct for multiple hypothesis testing. This method adjusts raw *P* values by scaling each value according to its rank and the total number of tests *m*. Refer to equation 5, where *m* is 6284 in our experiment, $p_{j}$ are raw *P* values, and the term *j* is the rank of the *P* value in ascending order. A reverse cumulative minimum is then applied to ensure monotonicity across the adjusted *P* values (equation 6), as described by Dudoit et al [30] and implemented in the *Statsmodels* package. These steps ensure that any day with an adjusted *P* value at or below .05 is considered a statistically significant anomaly, while the expected proportion of false discoveries is controlled. Importantly, the adjusted *P* values, $q_{k}$, are guaranteed to be nondecreasing with respect to the ordered raw *P* values.


(5) $${\tilde{p}}_{j}=min\left(\frac{m\times p_{j}}{j},1\right)$$

(6) $$q_{k}=min\left({\tilde{p}}_{j}\right)\;and\;j\geq k$$

### Ethical Considerations

The research protocol of the study described in this paper was approved by the institutional review board (IRB) of the University of Pittsburgh (study 20030193). As no patients were directly enrolled in this study and no compensation was provided, the IRB granted a waiver of informed consent. The NRDM dataset contains only aggregated purchase counts and does not include any individual-level purchase information. For the ED encounter data, a UPMC-approved honest broker deidentified the records to satisfy the requirements for a limited data set before distribution to our research team.

## Results

### Overview

This section presents the performance and behavior of NRDM Tracker. We begin by summarizing descriptive statistics of daily OTC product sales across 7 symptom-related categories (*Descriptive Statistics* section). Next, we describe the estimation of model parameters using the EM algorithm (*Model Parameters* section). We then evaluate the model’s ability to detect population-level anomalies during the COVID-19 pandemic using NRDM data (*Detection of COVID-19* section). Finally, we assess the statistical significance of daily deviations in product sales across individual categories to identify which product categories are influenced by a putative outbreak (refer to the *Detection of COVID-19* section).

### Descriptive Statistics

Table 3 lists descriptive statistics of the daily counts of the 7 categories from 274 stores in Allegheny County, PA from June 1, 2016, to December 30, 2021. The median of the daily OTC sales count over the 7 categories is 4062, with a wide range from 259 to 15,979, indicating substantial daily variation. The cough or cold category has the highest average daily sales (3168), accounting for most of the total OTC purchases recorded in NRDM. Moderate-sale categories include antifever for adult, baby or child electrolytes, and antidiarrheal. Antifever for pediatric, hydrocortisones, and thermometers have relatively low average daily sales. Most categories show right-skewed distributions (mean>median), suggesting occasional sales surges. The large variances (especially in total and cough or cold categories) indicate irregular and potentially seasonal demand.

**Table 3.** Summary statistics of daily over-the-counter (OTC) product sales across seven product categories obtained from the National Retail Data Monitor (NRDM) in Allegheny County, Pennsylvania, USA, from June 1, 2016, to December 30, 2021.

Category	Mean (SD)^a,b^	% of Total^a^	Median (IQR)	5% quantile^a^	95% quantile^a^	Min	Max
Cough or cold	3168 (1408)	71.8	2961 (1983‐4031)	1531	5652	172	12,149
Antifever for adult	503 (100)	12.7	495 (450‐546)	385	650	32	1422
Antifever for pediatric	121 (49)	2.9	112 (93‐136)	70	198	15	798
Baby or child electrolyte	152 (62)	3.8	139 (108‐187)	79	256	18	764
Thermometer	43 (33)	1	36 (26‐52)	17	88	0	734
Antidiarrheal	222 (41)	5.6	218 (195‐243)	165	291	15	433
Hydrocortisone product	83 (24)	2.2	79 (66‐97)	52	130	4	217
Total	4291	—^c^	—	—	—	—	—

a Percentages represent the proportion of total sales for each product category and are rounded to one decimal place; other values are rounded to integers.

b Total of all categories=4291.

c Not applicable.

Figure 2 illustrates seasonal patterns in OTC product sales over 5 years. Both total and “cough or cold” categories exhibit strong seasonal patterns, with recurring peaks during the winter months. A prominent spike in early 2020 likely corresponds to the onset of COVID-19, followed by a dip and gradual return to seasonal trends.

**Figure 2.**
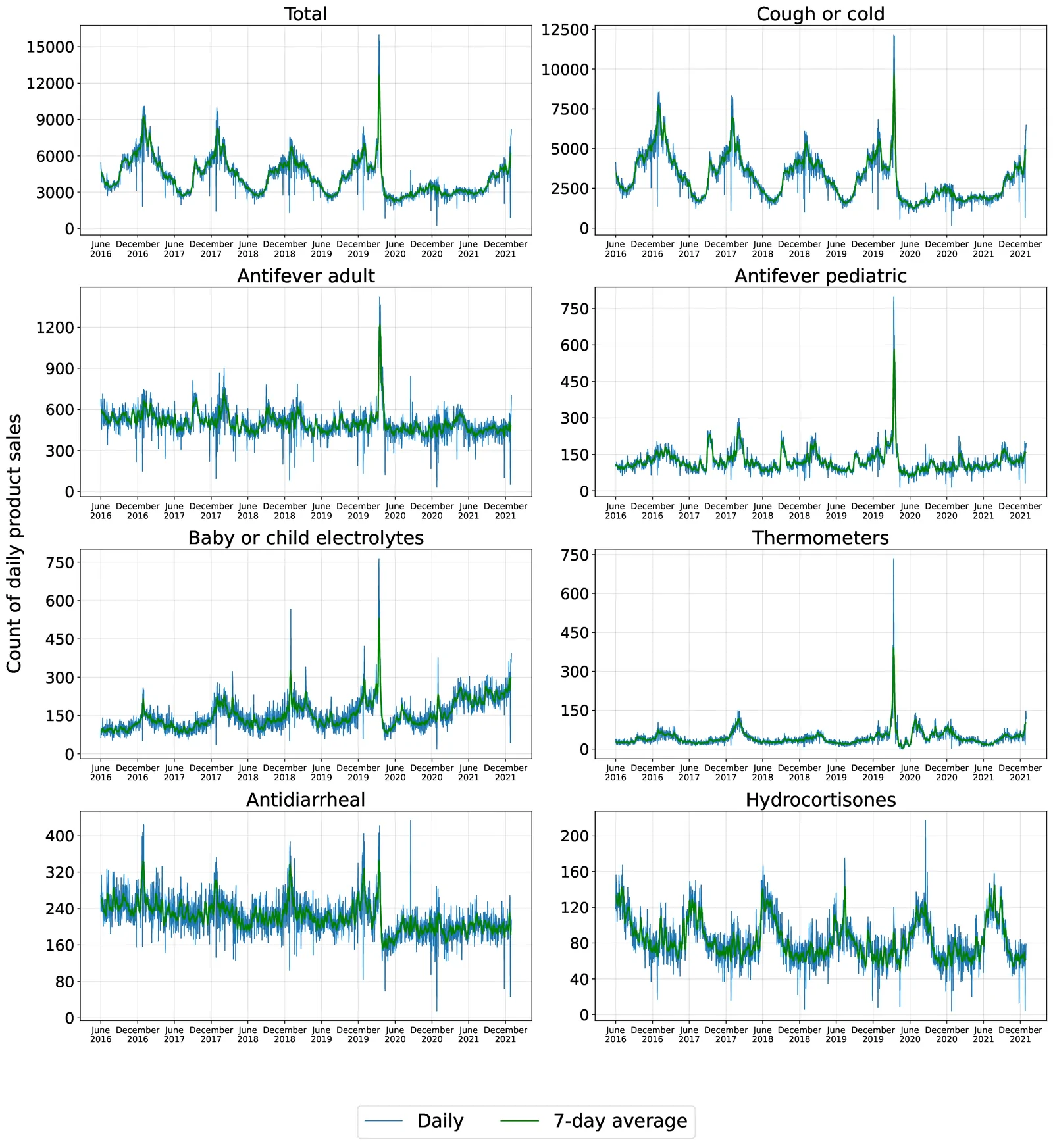
Temporal trends of daily over-the-counter (OTC) product sales recorded in the National Retail Data Monitor (NRDM) from 270 retail stores in Allegheny County, Pennsylvania, United States, from June 2016 to December 2021.

Sales trends among other product categories reveal varying degrees of seasonality and pandemic impact. Among antifever products, the adult antifever category shows relatively stable daily sales with modest fluctuations until a sharp surge in 2020, a pattern likely explained by the dual role of acetaminophen, ibuprofen, and related medications as both antipyretics and analgesics, which contributes to their steady baseline demand. The pediatric antifever category follows a similar seasonal trend but at a lower scale. Cough or cold products dominate in both volume and seasonality. Baby or child electrolytes show periodic increases with peaks in the winter. Antidiarrheal sales exhibit moderate daily variation with peaks around December. The peaks in hydrocortisone sales are less pronounced but show a consistent seasonal pattern, with increased use in warmer months, plausibly due to higher incidence of skin irritation, insect bites, and sun-related conditions. Thermometer sales remain low at baseline but exhibit dramatic, event-driven spikes—particularly in early 2020—likely due to increased public concern during the onset of the COVID-19 pandemic.

Figure 1 illustrates the temporal evolution of the estimated daily incidence rate of ILI (used as the prior probability in the model), derived from ED case counts for adenovirus, rhinovirus or enterovirus, influenza, HMPV, PIV, and RSV. The series exhibits clear seasonal peaks occurring annually—typically during the winter months (eg, early 2017, early 2018, spring 2019, and early 2020)—consistent with typical seasonal trends of respiratory viruses in this region. A pronounced decline is observed in 2020 and 2021, reflecting reduced ILI-related ED visits during the initial phase of the COVID-19 pandemic in the United States. A modest resurgence is seen in late 2021, coinciding with the return of seasonal respiratory virus activity.

### Model Parameters

Using the EM Algorithm, we estimated the conditional probabilities $P\left(product_{j}|ILI\right)$ and $P\left(product_{j}|OTHER\right)$. The EM algorithm converged after 36 iterations. Table 4 presents the estimated conditional probabilities, indicating the likelihood that each product category is purchased given an ILI-related or OTHER encounter. These values reflect how specific each product is to ILI activity. Cough or cold medications show a notably higher probability under ILI (0.792) than under OTHER (0.695), suggesting strong relevance to ILI-related symptoms. In contrast, the other products have lower probabilities for ILI than for OTHER, indicating they are more likely to be associated with non-ILI conditions. These conditional probabilities are central to inferring ILI activity from purchase patterns and help distinguish symptom-specific products from those with more general therapeutic use.

**Table 4.** Conditional probabilities estimated by the EM algorithm.

Category	$P\left(product_{j}|ILI\right)$ ^a^	$P\left(product_{j}|OTHER\right)$ ^a^
Cough or cold	0.792	0.695
Antifever for adult	0.095	0.142
Antifever for pediatric	0.024	0.031
Baby or child electrolyte	0.026	0.032
Thermometer	0.008	0.008
Antidiarrheal	0.043	0.064
Hydrocortisone	0.013	0.029

a Values are rounded to three decimal places.

To assess the robustness of the assumption of time-invariant conditional probabilities, we conducted a sensitivity analysis using time-varying conditional probability tables (CPTs) estimated with a rolling 12-month EM framework and weekly updates (Multimedia Appendix 3). As illustrated in Figure S1 and Table S2 in Multimedia Appendix 3, the estimated CPTs exhibit moderate temporal variation, reflecting changes in product use patterns and disease presentation over time. However, when these dynamic CPTs were incorporated into the NRDM-ILI estimation pipeline, the downstream impact was limited. The resulting NRDM-ILI time series closely matched those obtained using constant CPTs (Figure S2 in Multimedia Appendix 3), with nearly identical correlations with ED-ILI (0.6642 vs 0.6599). These findings suggest that, although conditional probabilities are not constant, the assumption of time-invariant CPTs is reasonably robust in this setting, and the primary conclusions of the model are not very sensitive to this assumption.

Although fever is a defining symptom of ILI, antifever medications show relatively lower conditional probabilities under ILI compared with cough or cold medications. This likely reflects differences in product use behavior rather than inconsistency with clinical definitions. Similar patterns are observed for other product categories, including those associated with ILI symptoms, where medications that are less specific to respiratory illness tend to show weaker associations. For example, thermometers, which are used for general fever monitoring rather than specifically for respiratory illness, show very low probabilities under both ILI and OTHER. These patterns are consistent across both the main analysis and the supplementary sensitivity analysis (Multimedia Appendix 3).

### Detection of COVID-19

Using equations 1 and 2, the NRDM Tracker calculated the expected counts of *ILI* from the NRDM data for the period from June 1, 2019, to December 30, 2021. The NRDM model was trained using data from June 1, 2016, to May 31, 2019, to estimate the CPTs; therefore, this latter period represents out-of-sample model performance.

Figure 3 illustrates the moving averages of those expected ILI counts in blue. The yellow curve shows *ILI* counts derived from the ED cases for the following viruses: adenovirus, rhinovirus or enterovirus, influenza, HMPV, PIV, and RSV, and shows strong temporal alignment with the curve in Figure 1, which depicts daily ILI priors. A disease caused by 1 of these viruses was considered present if a patient had a positive laboratory test result for the virus (eg, a positive PCR [polymerase chain reaction] test for influenza) or an *ICD* code indicative of the infection (listed in Multimedia Appendix 1). These ED-based ILI tests and diagnoses excluded COVID-19 cases, which emulates the situation in which a novel disease may occur before it is explicitly identified and tested for. The Pearson correlation coefficient between 2 time series shown in blue and yellow is 0.66.

**Figure 3.**
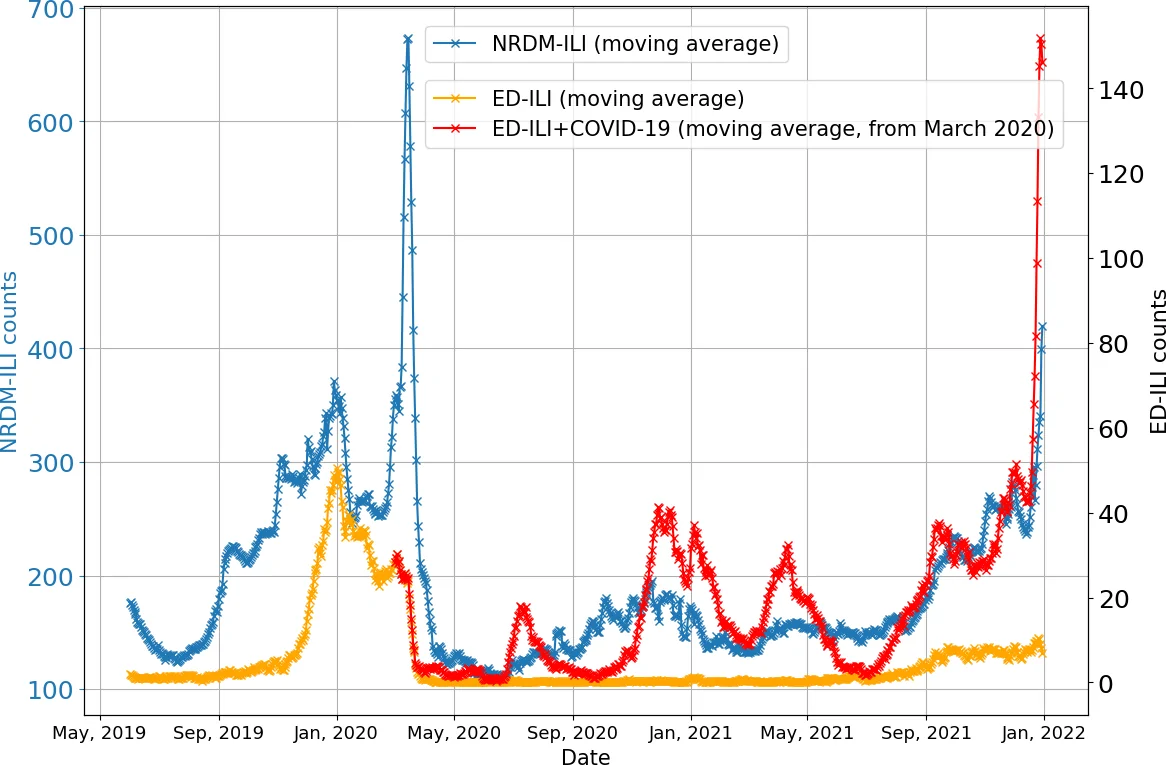
NRDM Tracker–estimated influenza-like illness (ILI) counts derived from over-the-counter (OTC) product sales (blue), emergency department (ED)–derived ILI counts based on positive lab*oratory* results and relevant *ICD* (*International Classification of Diseases)* codes for selected respiratory viruses (yellow; see text), and ED-derived counts that include both ILI and COVID-19 cases (red) in Allegheny County, Pennsylvania, United States, during the test period (June 2019-December 2021).

The peak day of the NRDM-estimated ILI counts is March 13, 2020 (based on the underlying data, although not perfectly aligned with the moving average curve in Figure 3), coinciding closely with the clinical onset of the COVID-19 in Allegheny County. Because NRDM-ILI reflects ILI activity inferred from consumer purchasing behavior, the peak on March 13 is likely due to precautionary purchasing triggered by widespread media coverage and growing public concern about COVID-19. The ILI counts from ED data have a peak on January 4, 2020 (not exactly aligned with the moving average curve), which corresponds to increased influenza and RSV activity observed in our ED data in late 2019 and in January and February 2020. A corresponding increase is seen in the NRDM signal during that period as well (Figure 3, blue curve).

In addition, Figure 3 includes a red curve representing ED-derived counts of ILI and COVID-19 beginning in March 2020, corresponding to the initiation of COVID-19 testing in Allegheny County. As COVID-19 subsequently spread throughout the county, it became the dominant respiratory infectious agent, while influenza and RSV cases declined sharply. Consequently, the NRDM-ILI trend (blue curve) began to align more closely with the ED-derived counts that include COVID-19 (red curve), rather than the ED counts that do not (yellow curve).

Figure 4 contains 2 plots displaying the daily log-likelihood of the OTC data computed by NRDM Tracker to monitor for unusual data patterns that may indicate the outbreak of a novel disease. The top panel shows the daily log-likelihood. A prominent drop is visible on March 13, 2020. Interestingly, the top panel also shows sharp declines on other dates, such as July 2020, suggesting potential anomalies in purchasing patterns. The bottom panel depicts the normalized daily log-likelihood, computed as the geometric mean of category-level probabilities. This measure indicates whether there are changes in the relative proportions of product category purchases. The normalized likelihood curve exhibits seasonal patterns and a sustained decline in mid-2020, followed by gradual recovery through 2021. The start of the COVID-19 outbreak does not appear to be associated with a strong change in the relative purchase of product categories, and thus, the prominent drop in the red curve in Figure 4 was driven by spikes or drops in total purchase counts rather than a shift in product mix.

**Figure 4.**
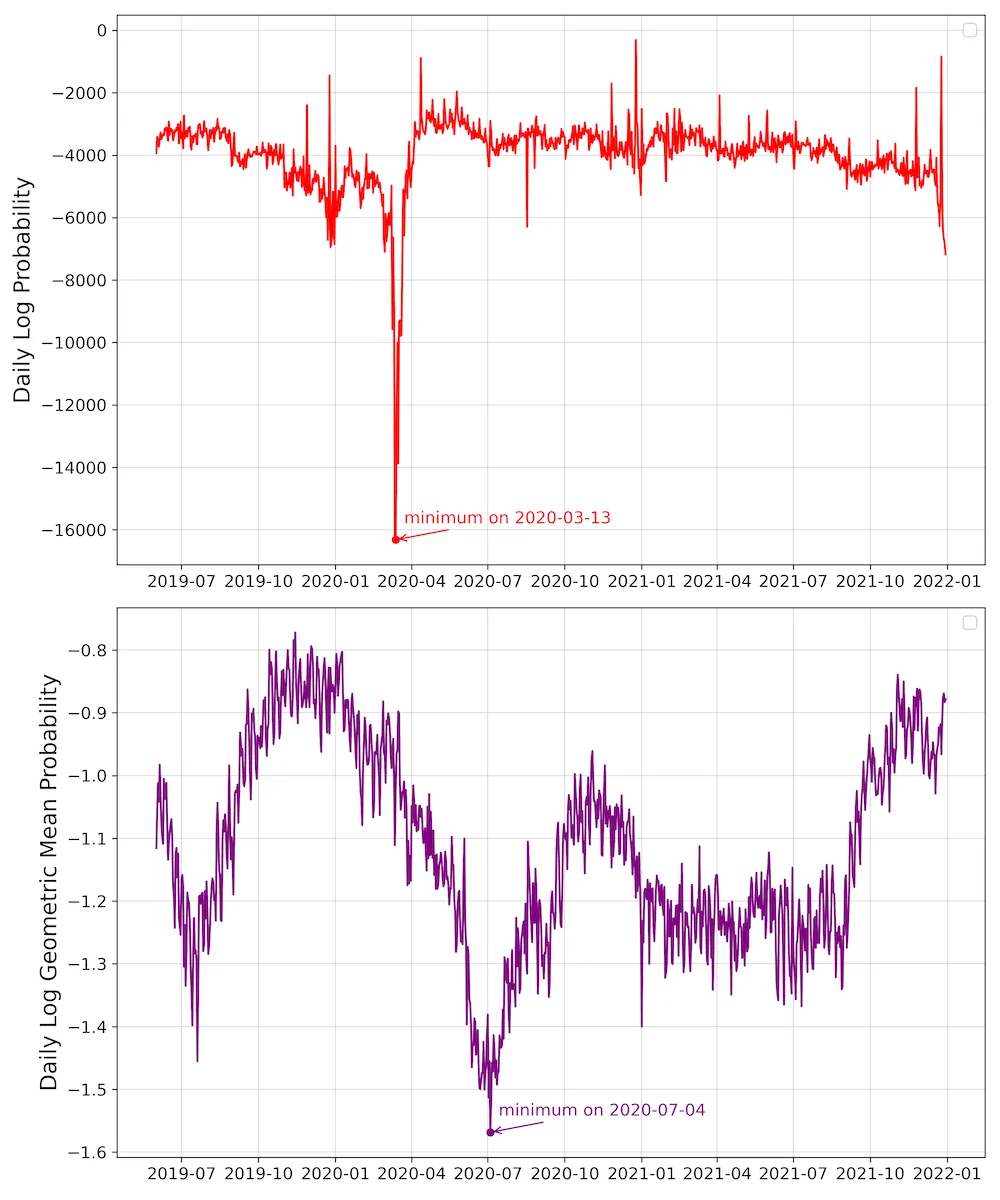
Daily log-likelihood of observed over-the-counter (OTC) purchasing patterns (top) and normalized log-likelihood (bottom) estimated by the NRDM tracker surveillance model using OTC product sales from Allegheny County, Pennsylvania, United States, during the test period (June 2019-December 2021). Lower values indicate greater deviation from expected purchasing patterns and may suggest unusual population-level health activity.

Using the Benjamini-Hochberg FDR procedure, we computed the adjusted *P* values for the daily counts of product sales for each of the 7 OTC categories. Figure 5 shows the time series of FDR-adjusted *P* values on a logarithmic scale, with the red dashed line indicating the significance threshold at *P*=.01. Sharp drops in *P* values—particularly around March 2020—are visible across all categories, reflecting significant deviations in product purchasing behavior during the early phase of the COVID-19 pandemic. Several other spikes and dips are also observed in late 2020 and late 2021, some of which may correspond to the resurgence of the COVID-19 pandemic due to evolving variants of SAR-CoV-2.

**Figure 5.**
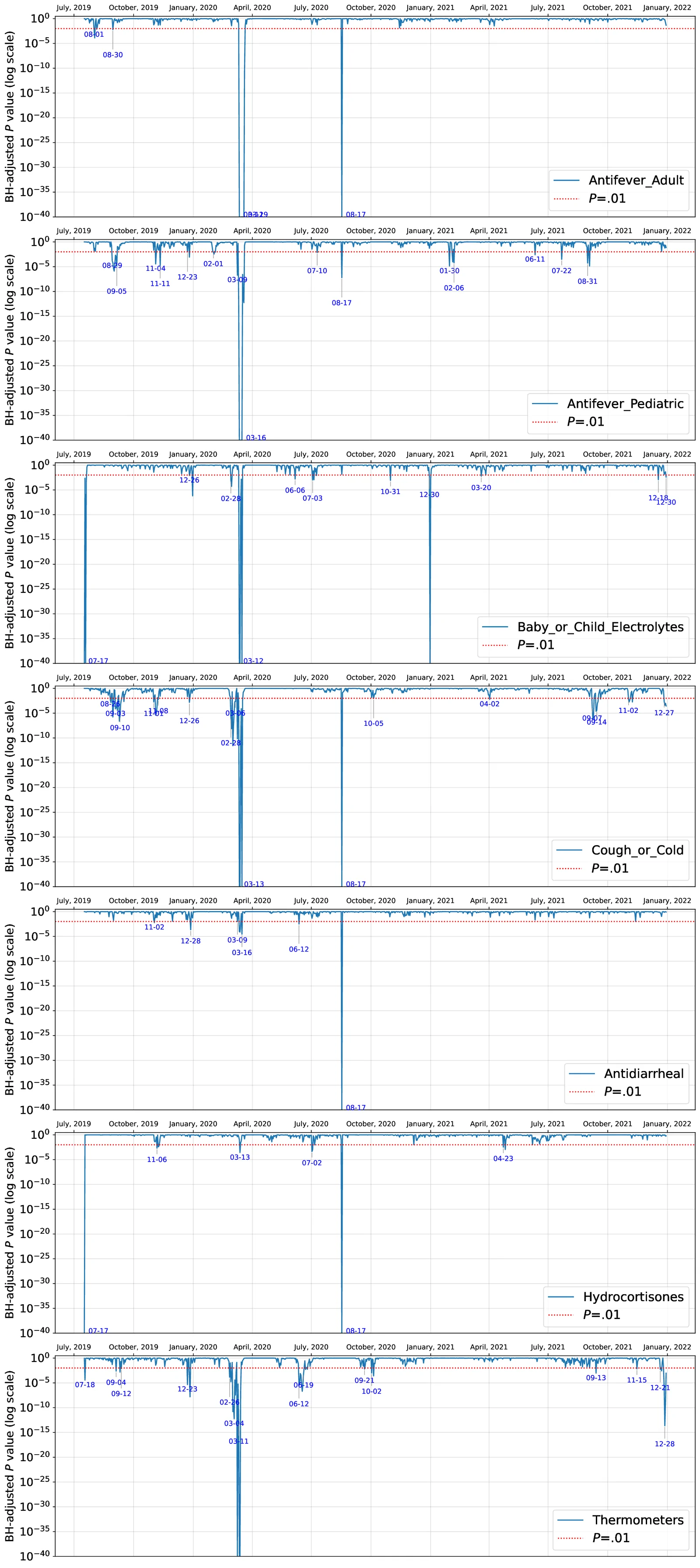
False discovery rate (FDR) adjusted *P* values for daily anomalies in over-the-counter (OTC) product sales across seven monitored product categories in Allegheny County, Pennsylvania, United States, during the test period (June 2019-December 2021). Values below the significance threshold (0.01) indicate statistically significant deviations from expected purchasing patterns detected by the NRDM Tracker system.

## Discussion

### Principal Findings

This paper investigates the potential for NRDM Tracker, using OTC data, as a tool for public health surveillance and early outbreak detection. By leveraging OTC product sales and applying a probabilistic modeling approach, we were able to estimate daily ILI activity and identify abnormal population-level purchasing patterns. The EM-based algorithm estimated conditional probabilities that distinguish ILI-related purchasing behavior from that associated with other health conditions. The resulting *ILI* estimates from NRDM data exhibited moderately strong correlation (*r*=0.66) with ILI-related ED visits, which provides support for the model’s alignment with measurable clinical rates of *ILI* disease in the region.

### Interpretation of Findings and Comparison With Previous Work

Our findings indicate that the NRDM-based ILI estimates, derived from OTC purchasing patterns, can capture public behavioral responses to disease outbreaks. These responses likely include purchases of cough and cold medications, antipyretics, and thermometers, even in the absence of corresponding clinical ILI encounters. Such dynamics could explain the divergence between the NRDM and ED curves in late 2019 and very early 2020, when NRDM estimates rose sharply while ED diagnoses for ILI remained comparatively stable. In general, while there is a close correspondence between the NRDM and ED curves in Figure 3, it is apparent that they are at times different, with an NRDM-derived estimate of increased ILI counts sometimes preceding the signal from ED data.

It is important to note that ED-based ILI surveillance, while widely used, may underestimate the true burden of ILI in the population [31], as many individuals with mild symptoms do not seek care in EDs. As a result, ED-derived ILI counts may primarily reflect more severe cases and may lag behind broader community-level disease activity. This limitation may help explain some of the observed differences between NRDM-based estimates and ED-based ILI signals. In particular, OTC purchasing behavior may capture earlier or milder symptom responses in the population, such as self-treatment before seeking professional medical care. A previous study [32] has noted temporal lags between community-level infection trends and subsequent increases in health care use, including ED visits and hospitalizations. In this context, the NRDM-based signals observed in this study may reflect earlier-stage or less severe disease activity, complementing ED-based surveillance. Together, these findings suggest that NRDM-based and ED-based estimates capture complementary aspects of disease activity, and that combining these signals may provide a more comprehensive assessment of population-level ILI than either source alone. Although fever is a defining symptom of ILI, antifever medications show a relatively weaker association with ILI activity compared with cough or cold medications in our analysis. This likely reflects differences in product use behavior rather than inconsistency with clinical definitions. Antifever medications are commonly used across a wide range of conditions and may already be available in households, reducing the need for purchase during illness episodes. In addition, OTC purchasing behavior may capture less severe cases of ILI, where individuals may not require or choose to take antipyretic medications, whereas ED-based surveillance is more likely to reflect more severe cases, potentially with higher fever. Similar patterns are observed for other general-purpose products, which tend to show weaker associations with ILI compared with more symptom-specific medications.

The NRDM Tracker system also derived daily likelihoods (in log form) of observed sales patterns as an overall measure of how well its model fit the OTC data. If the log-likelihood value on a given day is very low, relative to the values on previous days, it suggests the possibility of an outbreak. If so, those product categories with low *P* values are likely contributing to the low log-likelihood. Our results suggest that likelihood-based signals may provide an early indication of abnormal health-related activity. The sharp drop in log-likelihood observed in March 2020 aligns well with the initial COVID-19 outbreak in the United States. Importantly, our right-tailed *P* value framework—combined with multiple comparison correction—allowed for detection of statistically significant anomalies across multiple product categories. This analysis provides insights into the product sales that drive the detection of an anomaly.

Compared with previous studies that primarily examined associations between OTC sales and health care use [19,33] or evaluated NRDM for specific outbreak investigations [20,21,23,24], our study extends this body of work by developing a probabilistic surveillance framework capable of both estimating population-level ILI activity and identifying statistically significant anomalies that may indicate emerging or unexpected disease activity. Although previous studies have demonstrated that OTC purchasing data collected through NRDM can provide complementary syndromic signals of infectious disease activity [19,23,24], these studies have primarily focused on evaluating relationships with known clinical outcomes or detecting predefined outbreak events. In contrast, the NRDM Tracker presented in this paper uses probabilistic modeling of product category–specific purchasing patterns to continuously estimate ILI activity and detect statistically significant deviations from expected behavior, providing a framework that may support both seasonal disease monitoring and early identification of abnormal health-related signals potentially associated with emerging infectious disease activity.

### Limitations

There are several limitations of our approach. First, NRDM sales data do not directly record patient diagnoses, making it challenging to validate findings against gold-standard clinical data without assumptions. Second, behavioral factors may influence OTC purchasing patterns independently of underlying disease activity. For example, during the early phase of the COVID-19 pandemic, widespread public concern and media coverage likely contributed to “panic purchasing” of symptom-related products such as fever-reducing medications and thermometers, leading to transient spikes in sales that may not directly reflect true disease incidence. In addition, changes in supply chains, product availability, and public health advisories—both historically and prospectively—may alter purchasing behavior over time. Public statements or widely disseminated health communications, as for example, those related to the safety of specific medications, may also influence consumer behavior and health care–seeking patterns. These factors may introduce deviations in observed OTC signals that are not directly attributable to disease dynamics, potentially leading to false-positive signals of outbreaks. Finally, our modeling assumes stable conditional probabilities over time, which may not hold true under significant shifts in disease presentation or consumer behavior. This limitation could be attenuated by periodically updating the conditional probabilities.

Taken together, these limitations point to challenges that remain to be addressed in future work. Future research may also enhance the current work by incorporating additional data sources (eg, participatory surveillance systems [9,11,12], social media signals [34,35], and other digital epidemiologic data streams [36]) and by applying adaptive models that account for evolving behaviors and changing disease dynamics [37]. These directions are consistent with recent advances in AI [38] for integrating heterogeneous data sources to improve infectious disease surveillance and epidemic modeling [39].

### Conclusions

In summary, our results provide support that NRDM-based data, when paired with probabilistic modeling and anomaly detection techniques, may provide an effective supplement to traditional disease monitoring systems. In particular, the observed alignment between NRDM-derived ILI estimates and ED-reported ILI cases, along with the detection of known public health events (eg, COVID-19 onset), support the system’s potential for early detection of seasonal disease activity and for identifying anomalies that may be associated with novel disease outbreaks. By identifying significant deviations in symptom-related OTC product sales, the system may offer timely public health insights that could support outbreak monitoring and response. Continued refinement and integration with other data sources and modeling frameworks may further strengthen the role of OTC data in the national biosurveillance ecosystem.

## Supplementary material

10.2196/91163 Multimedia Appendix 1 *ICD* (*International Classification of Diseases*) diagnosis codes used to define influenza-like illness (ILI)–related diseases in emergency department (ED) surveillance data from Allegheny County, Pennsylvania, United States. The table includes *ICD-9* (*International Classification of Diseases, Ninth Revision*) and *ICD-10* (*International Classification of Diseases, Tenth Revision*) diagnosis codes used to identify adenovirus, enterovirus/rhinovirus, influenza, human metapneumovirus (HMPV), parainfluenza virus, respiratory syncytial virus (RSV), and COVID-19 cases for estimation of ILI activity in this study.

10.2196/91163 Multimedia Appendix 2 Expectation-maximization algorithm used to estimate OTC product purchase probabilities for influenza-like illness surveillance.

10.2196/91163 Multimedia Appendix 3 Sensitivity analysis using time-varying conditional probability tables estimated by the expectation-maximization (EM) algorithm.

## Abbreviations

CDC: Centers for Disease Control and Prevention
CPT: conditional probability table
E-step: expectation step
ED: emergency department
EHR: electronic health record
EM: expectation-maximization
FDR: false discovery rate
HMPV: human metapneumovirus
ICD: International Classification of Diseases
ILI: influenza-like illness
IRB: institutional review board
M-step: maximization step
NRDM: National Retail Data Monitor
OTC: over-the-counter
PCR: polymerase chain reaction
PIV: parainfluenza
RSV: respiratory syncytial virus
UPMC: University of Pittsburgh Medical Center
