# Supplementary material for: Using Over-the-Counter Retail Medication Sales to Detect and Track Influenza-Like Illnesses Including Novel Diseases: Surveillance Modeling Study
Source: JMIR Public Health Surveill. 2026 Oct 21;12:e91163. doi: 10.2196/91163 (PMC13686242; doi:10.2196/91163)
Supplement: Supplementary file 1 [file publichealth-v12-e91163-s001.docx]

**Appendix 1**

**Table S1. ICD diagnosis codes used to define influenza-like illness (ILI)–related diseases in emergency department (ED) surveillance data from Allegheny County, Pennsylvania, USA. The table includes ICD-9 and ICD-10 diagnosis codes used to identify adenovirus, enterovirus/rhinovirus, influenza, human metapneumovirus (HMPV), parainfluenza virus, respiratory syncytial virus (RSV), and COVID-19 cases for estimation of ILI activity in this study.**

| **disease** | **diagnosis codes** | **code descriptions** |
| --- | --- | --- |
| ADENOVIRUS | 79 | Adenovirus infection in conditions classified elsewhere and of unspecified site |
|  | 480 | Pneumonia due to adenovirus |
|  | B34.0 | Adenovirus infection, unspecified |
|  | B30.1 | Conjunctivitis due to adenovirus |
|  | B30.0 | Keratoconjunctivitis due to adenovirus |
|  | B97.0 | Adenovirus as the cause of diseases classified elsewhere |
| COV19 | U07.1 | COVID-19 |
| ENTEROVIRUS | 8.67 | Enteritis due to enterovirus nec |
|  | B34.1 | Enterovirus infection, unspecified |
|  | B97.10 | Unspecified enterovirus as the cause of diseases classified elsewhere |
|  | B97.19 | Other enterovirus as the cause of diseases classified elsewhere |
| HMPV | B97.81 | Human metapneumovirus as the cause of diseases classified elsewhere |
|  | J12.3 | Human metapneumovirus pneumonia |
|  | J21.1 | Acute bronchiolitis due to human metapneumovirus |
| INFLUENZA | 487 | Influenza with pneumonia |
|  | 487.1 | Influenza with other respiratory manifestations |
|  | 487.8 | Influenza with other manifestations |
|  | 488 | Influenza due to identified avian influenza virus |
|  | 488.01 | Influenza due to identified avian influenza virus with pneumonia |
|  | 488.02 | Influenza due to identified avian influenza virus with other respiratory manifestations |
|  | 488.1 | Influenza due to identified 2009 H1N1 influenza virus |
|  | 488.11 | Influenza due to identified 2009 H1N1 influenza virus with pneumonia |
|  | 488.12 | Influenza due to identified 2009 H1N1 influenza virus with other respiratory manifestations |
|  | 488.19 | Influenza due to identified 2009 H1N1 influenza virus with other manifestations |
|  | 488.81 | Influenza due to identified novel influenza A virus with pneumonia |
|  | 488.82 | Influenza due to identified novel influenza A virus with other respiratory manifestations |
|  | 488.89 | Influenza due to identified novel influenza A virus with other manifestations |
|  | J09.X1 | Influenza due to identified novel influenza A virus with pneumonia |
|  | J09.X2 | Influenza due to identified novel influenza A virus with other respiratory manifestations |
|  | J09.X3 | Influenza due to identified novel influenza A virus with gastrointestinal manifestations |
|  | J09.X9 | Influenza due to identified novel influenza A virus with other manifestations |
|  | J10.00 | Influenza due to other identified influenza virus with unspecified type of pneumonia |
|  | J10.01 | Influenza due to other identified influenza virus with the same other identified influenza virus pneumonia |
|  | J10.08 | Influenza due to other identified influenza virus with other specified pneumonia |
|  | J10.81 | Influenza due to other identified influenza virus with encephalopathy |
|  | J10.82 | Influenza due to other identified influenza virus with myocarditis |
|  | J10.83 | Influenza due to other identified influenza virus with otitis media |
|  | J10.89 | Influenza due to other identified influenza virus with other manifestations |
|  | J10.1 | Influenza due to other identified influenza virus with other respiratory manifestations |
|  | J10.2 | Influenza due to other identified influenza virus with gastrointestinal manifestations |
|  | J11.00 | Influenza due to unidentified influenza virus with unspecified type of pneumonia |
|  | J11.08 | Influenza due to unidentified influenza virus with specified pneumonia |
|  | J11.1 | Influenza due to unidentified influenza virus with other respiratory manifestations |
|  | J11.2 | Influenza due to unidentified influenza virus with gastrointestinal manifestations |
|  | J11.81 | Influenza due to unidentified influenza virus with encephalopathy |
|  | J11.82 | Influenza due to unidentified influenza virus with myocarditis |
|  | J11.83 | Influenza due to unidentified influenza virus with otitis media |
|  | J11.89 | Influenza due to unidentified influenza virus with other manifestations |
| PARAINFLUENZA | 480.2 | Pneumonia due to parainfluenza virus |
|  | J12.2 | Parainfluenza virus pneumonia |
|  | J20.4 | Acute bronchitis due to parainfluenza virus |
| RSV | 480.1 | Pneumonia due to respiratory syncytial virus |
|  | 79.6 | Respiratory syncytial virus (RSV) |
|  | 466.11 | Acute bronchiolitis due to respiratory syncytial virus (RSV) |
|  | B97.4 | Respiratory syncytial virus as the cause of diseases classified elsewhere |
|  | J12.1 | Respiratory syncytial virus pneumonia |
|  | J20.5 | Acute bronchitis due to respiratory syncytial virus |
|  | J21.0 | Acute bronchiolitis due to respiratory syncytial virus |
