# Supplementary material for: Using Over-the-Counter Retail Medication Sales to Detect and Track Influenza-Like Illnesses Including Novel Diseases: Surveillance Modeling Study
Source: JMIR Public Health Surveill. 2026 Oct 21;12:e91163. doi: 10.2196/91163 (PMC13686242; doi:10.2196/91163)
Supplement: Supplementary file 2 [file publichealth-v12-e91163-s002.docx]

**Appendix 2. Expectation-Maximization Algorithm Used to Estimate OTC Product Purchase Probabilities for Influenza-Like Illness Surveillance**

**Algorithm 1. Expectation-Maximization (EM) algorithm used to estimate conditional probabilities of over-the-counter (OTC) product purchases given influenza-like illness (ILI) and non-ILI disease categories from National Retail Data Monitor (NRDM) sales data.**

The robust implementation of the EM algorithm (Algorithm 2) extends Algorithm 1 by incorporating enhancements to improve numerical stability and convergence reliability. It refines the E-step by computing log-likelihoods and applying a stabilized posterior probability calculation, which mitigates issues related to numerical underflow. In the M-step, a smoothing constant $\mathcal{E}$ is introduced to ensure stable updates of the conditional probabilities, thereby avoiding zero estimates in scenarios with sparse data. These modifications enhance the algorithm’s robustness and practical applicability while preserving the core iterative structure of Algorithm 1—alternating between updating posterior probabilities and refining conditional probability estimates until convergence is reached.

In our experiments, we initialize $P({product}_{j}|ILI)$ using a uniform distribution across the seven products (i.e., each product is assigned a probability of 1/7). Similarly, $P({product}_{j}|OTHER)$ is initialized uniformly across all products. We set the smoothing constant ϵ to 1×10^−8^, and the convergence threshold for probability changes, *tol*, is also set to 1×10^−8^.

**Algorithm 2. Robust implementation of the Expectation-Maximization (EM) algorithm incorporating log-likelihood calculations and smoothing to improve numerical stability when estimating conditional probabilities from OTC product sales data.**
