# Supplementary material for: Using Over-the-Counter Retail Medication Sales to Detect and Track Influenza-Like Illnesses Including Novel Diseases: Surveillance Modeling Study
Source: JMIR Public Health Surveill. 2026 Oct 21;12:e91163. doi: 10.2196/91163 (PMC13686242; doi:10.2196/91163)
Supplement: Supplementary file 3 [file publichealth-v12-e91163-s003.docx]

**Appendix 3. Sensitivity Analysis Using Time-Varying Conditional Probability Tables Estimated by the Expectation–Maximization (EM) Algorithm**

To capture temporal variation in product–diagnosis relationships, we estimated **dynamic conditional probability tables (CPTs)** using a rolling-window Expectation–Maximization (EM) framework. Specifically, for each update time point, we constructed a **12-month rolling training dataset** ending at that time and applied the same EM algorithm (described in the main text) to estimate $P(\text{product}_{j}\mid\text{diagnosis})$for all products and diagnoses. The final CPTs from each window were retained as **time-specific estimates**, resulting in a sequence of CPTs that evolve over time.

Figure S1 presents the resulting time-varying conditional probabilities estimated using this rolling 12-month EM framework with weekly updates from June 2019 to December 2021. Each panel (A–G) corresponds to one product category: Antifever (Adult), Antifever (Pediatric), Baby/Child Electrolytes, Cough/Cold, Antidiarrheal, Hydrocortisones, and Thermometers. Within each panel, the red line represents ILI and the blue line represents OTHER diagnoses. The y-axis shows the estimated conditional probability, while the x-axis represents time. Selected time points with the largest week-to-week changes are annotated to highlight periods of notable shifts.

As shown in Figure S1, the conditional probabilities are **generally stable over time**, with gradual trends and occasional localized changes. Some products, such as Cough_or_Cold (panel D), consistently exhibit higher probabilities, while others (e.g., Thermometers and Hydrocortisones) show lower baseline values. Several products (e.g., Antifever Adult, Baby/Child Electrolytes, and Antidiarrheal) display noticeable shifts during specific periods (e.g., early to mid-2021), indicating that product–diagnosis relationships are not strictly constant over time. These results demonstrate that the dynamic CPT framework captures **moderate temporal variation** in product usage and disease presentation.

It is important to note that the CPT values shown in Figure S1 differ from those reported in the main manuscript, as the latter are estimated using the **full three-season dataset**, whereas the dynamic CPTs are derived from **rolling 12-month windows** and therefore reflect local, time-specific patterns rather than a single global estimate.

Table S2 summarizes the distribution of the dynamic CPT estimates over time and compares them with the corresponding three-season estimates. Overall, the **mean values of the dynamic CPTs are consistent with the three-season estimates**, indicating that the global CPT provides a reasonable approximation of the average product–diagnosis relationship. At the same time, the dynamic CPTs exhibit **moderate variability**, with relatively small standard deviations and narrow interquantile ranges for most product categories. While certain products (e.g., Baby/Child Electrolytes and Hydrocortisones) show more noticeable fluctuations, the variation remains constrained and centered around stable values.

Despite these observable differences in CPTs over time, their impact on downstream NRDM-ILI estimation is **limited**. As shown in Figure S2, incorporating dynamic CPTs leads to only modest changes in the estimated NRDM-ILI time series compared with the constant CPT approach. The two NRDM-ILI curves largely overlap across the study period, indicating that the overall signal is stable with respect to temporal variation in the CPTs. Small differences are observed during periods of rapid change, where the dynamic CPT-based estimates appear slightly more responsive; however, these deviations do not substantially alter the overall trends. This is further supported by the comparable correlations between NRDM-ILI and ED-ILI: constant CPT (0.6642), dynamic CPT (0.6599).

Overall, these findings indicate that while CPTs exhibit **moderate temporal variation**, the assumption of time-invariant conditional probabilities is **reasonably robust** for NRDM-ILI estimation in this setting. The dynamic CPT approach provides a flexible extension that better reflects temporal dynamics, but it does not materially change overall performance. Therefore, the constant CPT approach is retained in the main analysis for its simplicity and interpretability, while the dynamic CPT results serve as a **sensitivity analysis that supports the primary** findings described in the paper.

**Table S2. Summary statistics of dynamic conditional probability (CPT) estimates generated using a weekly rolling 12-month Expectation–Maximization (EM) framework applied to National Retail Data Monitor (NRDM) over-the-counter (OTC) product sales data from Allegheny County, Pennsylvania, USA (June 2019–December 2021). Dynamic CPT estimates are compared with the corresponding three-season CPT estimates used in the primary analysis.**

| Category | 3-season estimates | Dynamic estimates of conditional probabilities (weekly rolling 12-month window) | | | | | | |
| --- | --- | --- | --- | --- | --- | --- | --- | --- |
|  |  | mean | std | median | q05 | q95 | min | max |
| P(Antifever_Adult \| ILI) | 0.095 | 0.110 | 0.023 | 0.100 | 0.093 | 0.162 | 0.091 | 0.168 |
| P(Antifever_Pediatric \| ILI) | 0.024 | 0.030 | 0.004 | 0.029 | 0.025 | 0.036 | 0.025 | 0.043 |
| P(Baby_or_Child_Electrolytes \| ILI) | 0.026 | 0.041 | 0.012 | 0.035 | 0.033 | 0.071 | 0.033 | 0.073 |
| P(Cough_or_Cold \| ILI) | 0.792 | 0.744 | 0.049 | 0.772 | 0.625 | 0.775 | 0.621 | 0.776 |
| P(Antidiarrheal \| ILI) | 0.043 | 0.048 | 0.009 | 0.046 | 0.041 | 0.068 | 0.031 | 0.069 |
| P(Hydrocortisones \| ILI) | 0.013 | 0.015 | 0.006 | 0.014 | 0.011 | 0.030 | 0.008 | 0.031 |
| P(Thermometers \| ILI) | 0.008 | 0.012 | 0.004 | 0.012 | 0.008 | 0.020 | 0.008 | 0.026 |
| P(Antifever_Adult \| OTHER) | 0.142 | 0.158 | 0.010 | 0.156 | 0.147 | 0.177 | 0.139 | 0.178 |
| P(Antifever_Pediatric \| OTHER) | 0.031 | 0.033 | 0.001 | 0.032 | 0.031 | 0.036 | 0.031 | 0.036 |
| P(Baby_or_Child_Electrolytes \| OTHER) | 0.032 | 0.046 | 0.008 | 0.044 | 0.039 | 0.067 | 0.038 | 0.067 |
| P(Cough_or_Cold \| OTHER) | 0.695 | 0.654 | 0.020 | 0.657 | 0.621 | 0.677 | 0.618 | 0.686 |
| P(Antidiarrheal \| OTHER) | 0.064 | 0.067 | 0.002 | 0.067 | 0.062 | 0.070 | 0.060 | 0.070 |
| P(Hydrocortisones \| OTHER) | 0.029 | 0.029 | 0.003 | 0.029 | 0.023 | 0.033 | 0.021 | 0.034 |
| P(Thermometers \| OTHER) | 0.008 | 0.013 | 0.005 | 0.011 | 0.007 | 0.019 | 0.007 | 0.021 |

*rounded to three significant digits

**Figure S1. Dynamic estimates of** $\boldsymbol{P}\left( \text{product} \mid\text{diagnosis} \right)$ **obtained using a rolling 12-month EM framework with weekly updates (June 2019–December 2021). Each panel corresponds to one OTC product category. Red lines denote ILI and blue lines denote OTHER diagnoses. Selected time points with the largest week-to-week changes are annotated.**

**Figure S2. Comparison of NRDM-derived influenza-like illness (ILI) estimates generated using constant and dynamically updated conditional probability tables (CPTs) during the test period (June 2019–December 2021), together with ED-derived ILI counts. Dynamic CPTs were estimated using a rolling 12-month EM framework with weekly updates. All time series were smoothed using a 7-day moving average. The two NRDM-ILI curves show similar temporal patterns with only minor differences.**
